# Early mobilization with or without cycloergometry in patients with septic shock in Intensive Care Unit: a randomized controlled trial

**DOI:** 10.1016/j.aicoj.2026.100034

**Published:** 2026-02-20

**Authors:** B. Michaux, V. Harter, E. Occhiali, A. Astier, A. Freynet, G. Fossat, R. Galliot, G. Mourrisoux, J. Charpentier, B. Rozec, F. Tamion, G. Béduneau, the Muevelo study group

**Affiliations:** a Physiotherapist Intensive Care Units Rouen Universitary Hospital, F-76000 Rouen, France; b North-West Canceropole Data Center (V Harter MSc), Baclesse Comprehensive Cancer Center, Caen, France; c Rouen University Hospital, Department of Anaesthesiology, Critical Care and Perioperative Medicine, Rouen, France; d Physiotherapist, Reanimation Neurologique et Centre Expert SLA, Hôpital Neurologique Pierre Wertheimer, Bron, France; e Physiotherapist, CHU de Bordeaux, Service d’Anesthésie-Réanimation, F-33600 Pessac, University Bordeaux, CNRS, Inserm, Immuno ConcEpT, UMR 5164, F-33000 Bordeaux, France; f Kinésithérapeute en Recherche Clinique, Phd Student SSMMH, UR 20201 ERPHAN Médecine Intensive et Réanimation, Centre Hospitalier Universitaire d’Orléans; g Réanimation Polyvalente, Hôpital Foch Suresnes, France; h CHU Bordeaux, Services de Réanimation Médicale, France; i Médical Intensive Care Unit, Cochin University Hospital, Hôpitaux Universitaires-Paris Centre, Assistance Publique-Hôpitaux de Paris, Paris, France; j Nantes Université, CHU Nantes, CNRS, INSERM, l’Institut du Thorax, F-44000 Nantes, France; k Univ Rouen Normandie, Inserm, Normandie University, ENVI UMR 1096, CHU Rouen, Department of Medical Intensive Care, F-76000 Rouen, France; l University Rouen Normandie, Normandie University, GRHVN UR 3830, Intensive Care Medicine Rouen Universitary Hospital, F-76000 Rouen, France

**Keywords:** Sepsis, Mechanical ventilation, Physiotherapy, Cycloergometry, Intensive care unit

## Abstract

**Purpose:**

Acquired weakness in intensive care unit (ICU) contributes to increased mechanical ventilation (MV) and morbi-mortality. Cycloergometry could be added to standard physiotherapy (SP). The objective of the study was to determine the effect of early mobilization with cycloergometry and SP on ICU length of stay (LOS).

**Methods:**

This prospective, randomized, multicenter study included sedated-ventilated patients admitted to ICU for septic shock. ICU LOS was assessed in two phases. Phase I: from hemodynamic stability to first awakening, and phase II: from first awakening to ICU discharge. In each phase, patients were randomized to an intervention group: cycloergometry and SP (C+SP), or a control group (SP), generating four groups in total. In the intervention group, patients received a daily session of 20 min of cycloergometry in addition to SP.

**Results:**

From December 2016 to March 2020, 119 patients were included (instead of 234 planned). Mean SAPSII score at ICU admission was 59.5. Characteristics at baseline were similar. When phase I and phase II were analyzed separately, no statistical difference was observed between groups in time to first awakening or time to ICU discharge (phase I: C+SP 4 [IQR 2–7] days vs SP 4 [IQR 2–8] days, p = 0.6, and phase II: C+SP 9 [IQR 6–15] days vs SP 12 [IQR 6–28] days, p = 0.3).

In post-hoc analysis when phase I and phase II were considered together, the median duration of MV was significantly longer in patients who received no cycloergometry (14 [IQR 8–60] days vs 9 [IQR 6–17] days, p = 0.04). Moreover, a trend was observed for a shorter time from hemodynamic stability to ICU discharge in patients who received cycloergometry in at least one phase: C+SP in phase I and/or phase II (13 [IQR 8–33] days) vs SP only (20 [IQR 11–66] days), p = 0.052.

**Conclusion:**

Although the planned number of patients could not be included, a non-significant signal for decreased ICU LOS was observed in patients who received cycloergometry in at least one phase, in post-hoc analysis. Furthermore, cycloergometry was associated with a significantly lower duration of artificial ventilation.

## Background

Septic shock is a life-threatening condition caused by a dysregulated host response to infection [1]. It is a major healthcare concern, affecting millions of people worldwide, with a mortality ranging from 15 to 37% [2]. Hospitalization in intensive care unit (ICU) exposes patients to many potentially serious outcomes, among them acquired weakness is one of the most difficult to manage, with an overall prevalence of 48% according to a recent meta-analysis [3].

Acquired weakness in ICU (ICU-AW) is caused by prolonged bed rest and multi-organ failure due to septic shock, and is responsible for an increase in the duration of mechanical ventilation and hospitalization, as well as higher morbidity and mortality [4]. In order to limit the effects of this neuromuscular dysfunction, physiotherapy is recommended in ICU by several medical societies [5,6]. Several observational studies [[7], [8], [9]] reported data on the feasibility and safety of early mobilization (EM). According to Schweickert et al. [10], early therapy improved outcomes such as independent walking at hospital discharge, ventilator-free days, and duration of delirium.

Cycloergometer sessions may be added to standard physiotherapy. Several studies have shown that in-bed cycling is safe for critically ill patients [[11], [12], [13], [14]]. We postulated that cycloergometry, from the hemodynamic stability of patients to ICU discharge, could provide benefits for these patients, by limiting muscle weakness, accelerating weaning from mechanical ventilation, and shortening ICU stay.

The aim of this study was to determine the effect of early mobilization, with cycloergometry and standard physiotherapy, on the length of stay in ICU of patients with septic shock.

## Methods

### Patients

This prospective, randomized, multicenter study recruited patients hospitalized in eight ICUs in France with a diagnosis of septic shock of more than 24 h before inclusion [15]. Patients had to be hemodynamically stable within 72 h following the diagnosis of septic shock, defined by a mean arterial pressure (MAP) ≥65 mmHg with no increase in vasopressors for at least 6 consecutive hours. Patients were included if they were mechanically ventilated with tracheal intubation, sedated with a Richmond agitation-sedation scale (RASS) score [16] ≤ −2, age ≥ 18 years, and a body mass index (BMI) ≤ 40 kg / m². Patients were not included if they were pregnant or breastfeeding, had neuro or spinal cord injury, were moribund or had a decision of limitation of life-sustaining care, had a contraindication to performing the protocol, had Extra Corporeal Membrane Oxygenation, were included in another interventional trial with the same objective, were deprived of their liberty, or were under guardianship/curatorship. All included patients or their legal representative provided written informed consent, and all trial procedures conformed to local policies. Results were partially presented as an abstract [17].

### Study procedure

ICU length of stay (LOS) was assessed in two phases. In each phase, patients were randomized to an intervention group, cycloergometry and standard physiotherapy (C+SP), or a control group (SP), generating four groups in total. Randomization was performed using an unblinded blocked randomization list. In phase I, patients were randomized at hemodynamic stability. In phase II, patients were randomized at first awakening (defined by a RASS score between −1 and +1). In the control group, patients received a daily session (excluding weekends) of SP (details in appendix). In the intervention group, patients received a daily session (excluding weekends) of C+SP of 20 min of cycloergometry in addition to standard physiotherapy. A completed physiotherapy session was defined as the performance, during the same day (Monday to Friday), of the planned physiotherapy activities (mobilizations +/− cycloergometry). This session was passive, active or resisted depending on the state (cognitive and muscular) of the patient. In the two groups, patients also received respiratory physiotherapy sessions, once or twice a day and 7 days a week if the patient's condition required it.

The physiotherapy activities included mobilizations, sitting over the edge of the bed, sitting in a chair, walking, and cycling, and were adapted to the patient's condition (cardiorespiratory, cognitive, muscular, etc.). There was no minimum duration of SP. In case of an adverse event (hypotension, tachycardia, polypnea, fatigue, pain, etc.), sessions were stopped or not started (details in appendix). A completed session corresponded to +/− 20 min of cycloergometry. Otherwise, we noted that sessions were stopped (<20 min of cycloergometry, mobilizations in bed stopped, failure to sit over the edge of the bed, etc) or not performed (no activity performed during the day).

The care of patients on all other levels (weaning from mechanical ventilation, medical care, nursing care, etc.) was left to the discretion of the centers following current recommendations. Investigators were therefore asked to extubate patients after successful weaning trials and to discharge them from ICU in the absence of artificial life support.

The local Institutional Review Board (Comité Protection Personnes Nord-Ouest 1; May 3, 2016) approved the study protocol. We did not plan a Data Safety Monitoring Board due to the absence of major risk associated with physiotherapy, with or without cycloergometry. Safety data were communicated once a year in accordance with French regulations (article R1123-61 du Code de la santé publique).

Trial registration: ClinicalTrials.gov (NCT02872792) registered 16 August 2016 and RCB number (2016-A00060-51).

### Primary endpoint

The primary endpoint was ICU LOS considered as the time from hemodynamic stability to ICU discharge.

### Secondary endpoints

Secondary endpoints were: (i) time from hemodynamic stability to first awakening (phase I), (ii) time from first awakening to ICU discharge (phase II) (iii) duration of mechanical ventilation (MV) (invasive and non-invasive), (iv) time from first awakening to first successful extubation (defined by the absence of reintubation within 48 h of extubation), (v) muscle strength calculated by the Medical Research Council (MRC) score [18], (vi) functional independence calculated by physical function in intensive care test score (PFIT-s) [19], (vii) hospital LOS.

### Statistics

The study was conducted in two phases: passive mobilization during phase I, and mostly active mobilization during phase II. As mortality during phase I was greater than during phase II [20], this may have influenced the effectiveness of the cycle ergometer. In order to minimize the risk of an imbalance between treatment arms, due to high mortality during phase I when patients were sedated, the primary endpoint was planned to be analyzed independently by phase with a randomization before each phase. This double randomization had the additional advantage of maintaining the physiotherapist in charge of passive mobilization during phase I blinded to the use of the cycle ergometer or not during phase II. The sample size was calculated to ensure a power of 80% and an overall alpha risk of 5% for the evaluation of the primary endpoint independently in phase I and phase II. Assuming a mortality of 20% in phase I and a mortality of 5% in phase II, the inclusion of 234 patients in phase I should make it possible to detect a subdistribution hazard ratio (SHR) of at least 1.60 in each phase between C+SP and SP groups. The double randomization constituted a dataset for the endpoint analysis in phase I and another dataset for unbiased endpoint analysis in phase II. It also defined four subgroups of patients for post-hoc analyses of the primary endpoint over the two phases. As time-to-event secondary endpoints could not be defined by phase, their pre-specified analyses were also based on these four subgroups. Evaluations performed on combinations of these subgroups were considered as post-hoc analyses.The primary endpoint was a time-to-event endpoint. As mortality seemed non-negligible and different between phase I and phase II, we used regression models integrating the notion of competing risk to estimate and test the effect of early mobilization by cycloergometry. Thus, we described the durations per treatment arm for phase I and for phase II by calculating the cumulative frequencies according to the Aalen model [21] and non-parametric Gray tests. We quantified the SHR between the two arms with 95% confidence intervals in a Fine and Gray regression model for competing risks [22] on bivariate analysis and also on multivariate analysis adjusted for clinical factors and scores (Sequential Organ Failure Assessment (SOFA), RASS, MRC and PFIT) recorded before each randomization. We used corrected Akaike information criterion (AICc) to select step-by-step the best-fitting proportional subdistribution hazard multivariate models with covariates of arms forced in the model.

Time-to-event secondary endpoint analyses involving mortality as a competing risk were performed using the same statistical methods: time from first awakening to first successful extubation, duration of mechanical ventilation (invasive and non-invasive) and hospital LOS.

To measure a direct association between the use of cycloergometry and overall survival from hemodynamic stability to ICU discharge, we fitted a Cox proportional hazard regression with arms at first and second randomization as a time-dependent covariate. Patients who did not meet inclusion criteria for the second randomization were censored at first awakening.

We described the other endpoints using standard descriptive statistical methods. Qualitative data are reported as frequency and percentage, quantitative data as either mean and standard deviation or as median and interquartile range (IQR) depending on the normal distribution assessment of the variable. We compared baseline characteristics with Student T-test, Wilcoxon-Mann-Whitney test, Chi square test or exact Fisher test depending on whether their statistical assumptions were met.

All patients were analyzed according to the intention-to-treat principle. No imputation of missing data was carried out. For each phase, all patients meeting the inclusion criteria and randomized were included in the analysis according to their arm regardless of the physiotherapy finally performed. For the combined post-hoc analysis, the four patient subgroups were defined according to the same principle but patients who did not reach the second randomization were excluded.

All statistical analyses were performed with R statistical software v4.0.2 (R Core Team (2020). R: A language and environment for statistical computing. R Foundation for Statistical Computing, Vienna, Austria).

## Results

### Characteristics of the population

**Fig. 1:**
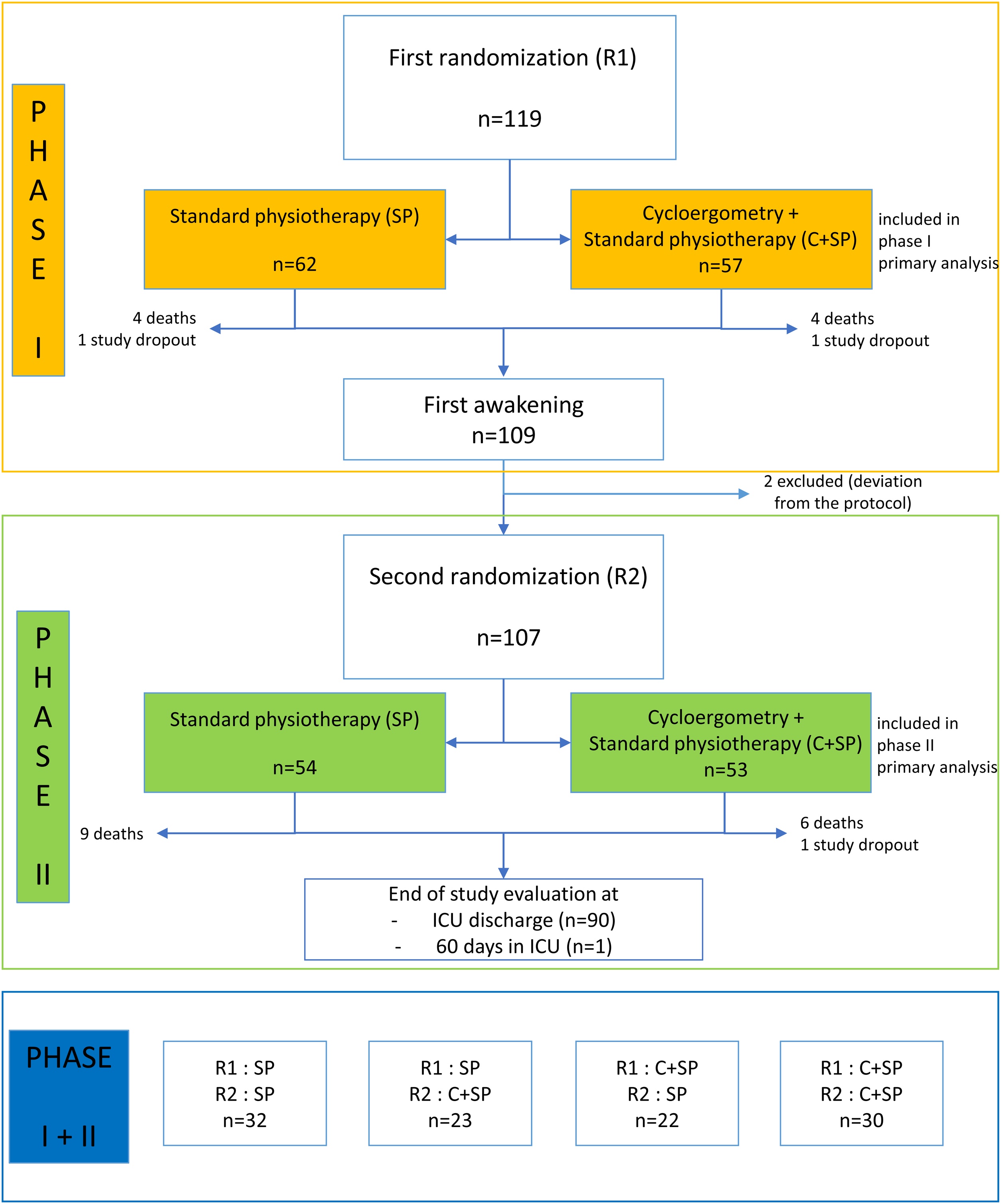
Flow chart of the study. ICU = intensive care unit.

From December 2016 to March 2020, 119 patients with a mean age of 65 years (±13) were included in the study. The target of 234 patients resulting from the initial enrolment calculation could not be reached due to a lack of eligible patients. The mean SAPS II and the median SOFA scores of patients at ICU admission were 59.5 (±18.7) and 9 [IQR 7−12], respectively. Among these 119 patients, 63 (53%) were admitted for medical reasons and 56 (47%) following surgery. The site of infection was mainly pulmonary (45%) or abdominal (45%) (Table 1). Patients’ characteristics at baseline were similar in the two groups in phase I (Supplementary Table S1) and in phase II (Supplementary Table S2). The randomization of patients in phase I and in phase II is presented in the study flow chart (Fig. 1). This double randomization generated four subgroups, one of which received no cycloergometry. Of note, in phase I, three patients received no physiotherapy (one patient in the SP group and two patients in the C+SP group). In phase II, all patients received at least one physiotherapy session.

**Table 1:** Baseline characteristics of the population included in phase I (n = 119).

		N = 119
Demography		
Age (yr)	Mean (SD)	65.2 (13.0)
Sex	Male	76 (64%)
	Female	43 (36%)
Weight (kg)	Mean (SD)	77.4 (19.1)
BMI (kg/cm²)	Mean (SD)	27.02 (5.84)
Admission to hospital and ICU		
Type of admission	Scheduled surgery	12 (10%)
	Urgent surgery	44 (37%)
	Medical	63 (53%)
Site of infection	Pulmonary	53 (45%)
	Abdominal	54 (45%)
	Urinary tract	8 (6.7%)
	Other	4 (3.4%)
Time between admission to ICU and hemodynamic stability	Less than 12 h	9 (7.6%)
	12 to 24 h	32 (27%)
	24 to 48 h	50 (42%)
	48 h to 72 h	28 (24%)
SAPS II score at admission to ICU	Mean (SD)	59.5 (18.7)
SOFA score at admission to ICU	Median (IQR)	9 (7-12)
RASS score before randomization	−2	4 (3.4%)
	−3	23 (19%)
	−4	36 (30%)
	−5	54 (45%)
	Unknown	2 (1.7%)

SP = standard physiotherapy. C+SP = cycloergometry + standard physiotherapy. BMI = body mass index. ICU = intensive care unit. SAPS = simplified acute physiology score. SOFA = sepsis-related organ failure assessment. RASS = Richmond agitation-sedation scale. SD = standard deviation. IQR = interquartile range.

For different reasons, including changes in practices between the start of the project and the inclusion period, the COVID pandemic which stopped inclusion in March 2020, and funding and insurance issues, we stopped the study in December 2020 without having reached the number of subjects calculated for optimal statistical analysis.

### Primary outcome

**Fig. 2:**
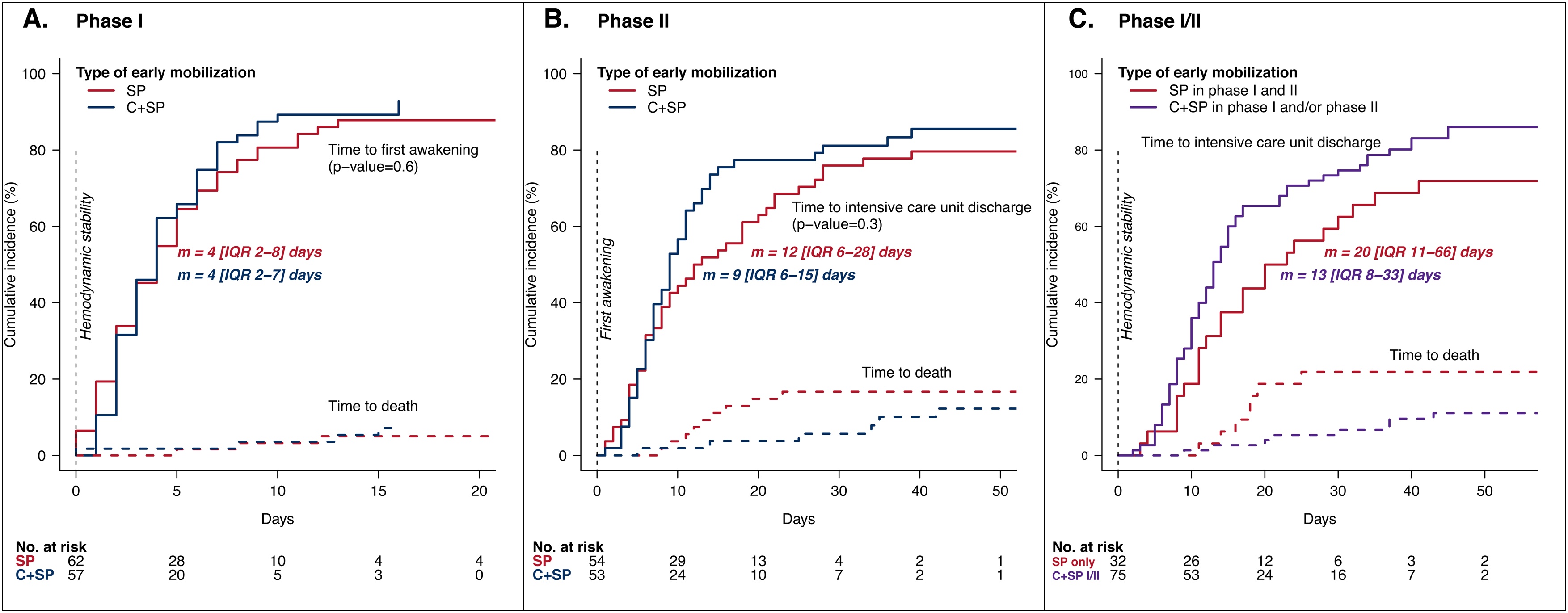
Intensive Care Unit stay in phase I, phase II and phase I/II. Results of Aalen cumulative incidence of first awakening from hemodynamic stability by arm for the 119 patients randomized in phase I (A, full lines); cumulative incidence of intensive care unit (ICU) discharge from first awakening by arm for the 107 patients randomized in phase II (B, full lines); cumulative incidence of ICU discharge from hemodynamic stability (phase I /II) by arm combination for the 107 patients randomized in phases I and II (C, full lines). For each curve, median duration and IQR are displayed in days. Cumulative incidence of deaths (competing event in Aalen model) are plotted in dashed lines. Number of patients at risk at various times is indicated under the x-axis. P-values result from Aalen model. SP = standard physiotherapy. C+SP = cycloergometry + standard physiotherapy. m = median. IQR = interquartile range.

When phase I and phase II were analyzed separately, no statistical difference was observed between groups in time to first awakening or time to ICU discharge (phase I: C+SP 4 [IQR 2–7] days vs SP 4 [IQR 2–8] days, p = 0.6, and phase II: C+SP 9 [IQR 6–15] days vs SP 12 [IQR 6–28] days, p = 0.3) (Fig. 2A, 2B). In multivariate analysis, adjusted for SOFA score at hemodynamic stability, we observed no evidence of an effect of mobilization with cycloergometry on time to first awakening in phase I (SHR = 1.10; 95% CI 0.79–1.54, p = 0.6), or on time to ICU discharge in phase II, (SHR = 1.50; 95% CI 0.83–2.72, p = 0.18) after adjusting for type of mobilization in phase I, SOFA score at first awakening and MRC score at first awakening (Supplementary Table S3).

When phase I and phase II were considered together in post-hoc analysis, a trend was observed for a shorter time from hemodynamic stability to ICU discharge in patients who received cycloergometry in at least one phase: C+SP in phase I and/or phase II (13 [IQR 8–33] days) vs SP only (20 [IQR 11–66] days), p = 0.052) (Fig. 2C, Supplementary Fig. S1A).

### Secondary outcomes

**Fig. 3:**
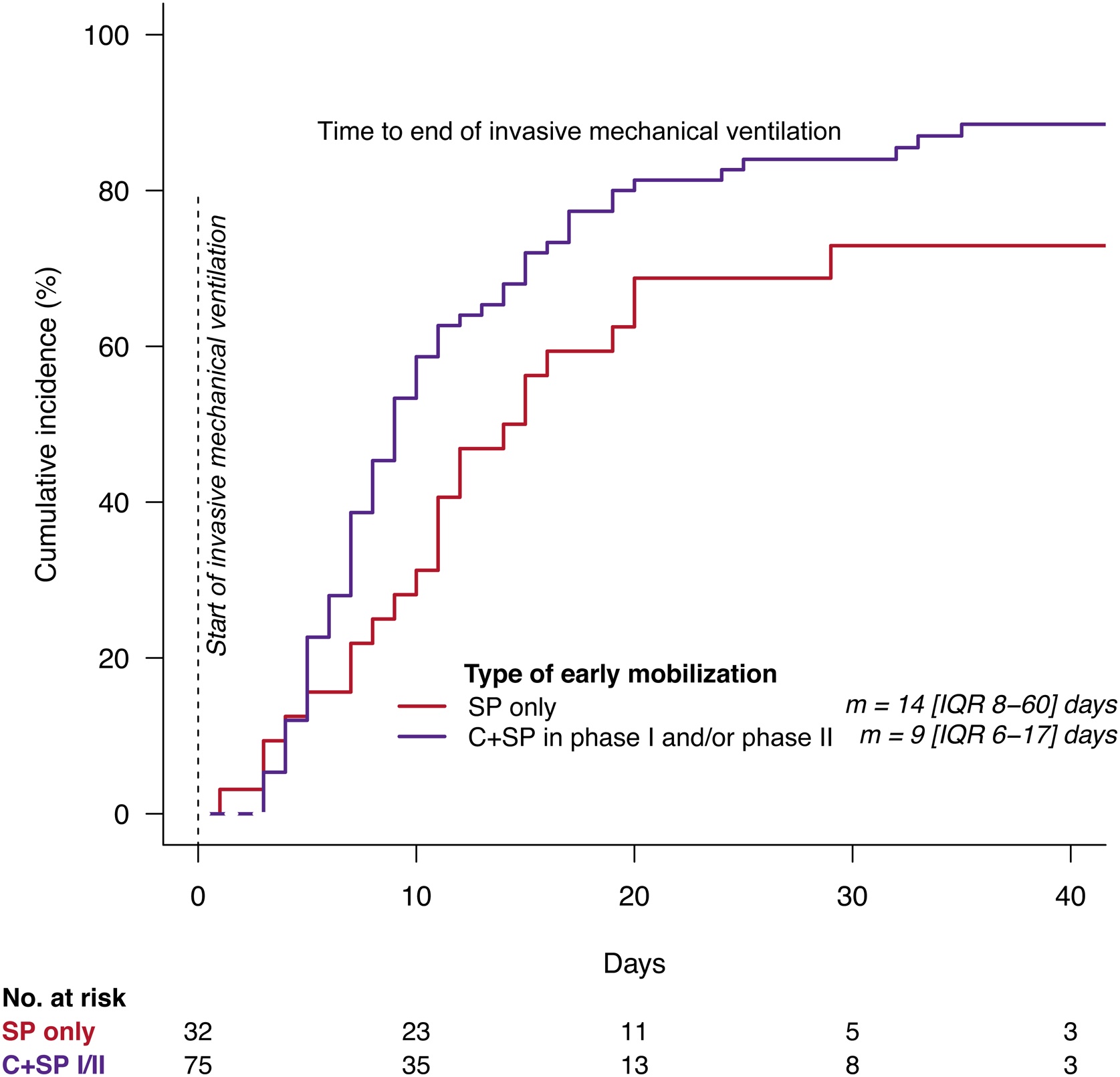
Duration of invasive mechanical ventilation SP vs others. Results of Aalen cumulative incidence of stopping invasive mechanical ventilation (IMV) by arm combination (SP only vs C+SP in phase I and/or phase II) for the 107 patients randomized in phase I and II. Median duration and IQR are displayed in days. The number of patients at risk at various times is indicated under the x-axis. P-value results from Aalen model. SP = standard physiotherapy. C+SP = cycloergometry + standard physiotherapy. m = median. IQR = interquartile range.

The median duration of invasive MV was significantly longer in patients who received no cycloergometry in any phase (14 [IQR 8–60] days vs 9 [IQR 6–17] days, p = 0.04) (Fig. 3). When analyzing the four subgroups, we did not observe an effect of early mobilization on the duration of invasive MV in phase I, phase II or phase I/II (p = 0.18) (Supplementary Fig. S2). The observations were similar when invasive MV and non-invasive MV were considered together (Supplementary Fig. S3). At ICU discharge, no statistical difference was observed between groups in MRC score (SP: 48 [IQR 38–54] vs C+SP: 48 [IQR 42–55.5]) or PFIT score (SP: 6 [IQR 4–8] vs C+SP: 7 [IQR 4–8.5]). MRC and PFIT scores at first awakening are presented in Supplementary Table S4 (missing data).

No significant difference was observed in the other parameters: hospital LOS (Supplementary Fig. S1B) or time from first awakening to first successful extubation (Supplementary Fig. S4).

### Security and feasibility

Physiotherapy sessions were started at a median of 2 [IQR: 2–3] days after ICU admission in the two groups (Supplementary Table S5).

The number of physiotherapy sessions performed was similar in the two groups: in phase I, three sessions [IQR 2–5] in the SP group and three sessions [IQR 2–4] in the C+SP group, and in phase II, seven sessions [IQR 4–12] in the SP group and six sessions [IQR 3–9] in the C+SP group.

Moreover, in phase I more than 90% of patients had at least one session of each activity described (mobilization +/− cycloergometry). Mobilization and cycloergometry were performed in more than 75% of the sessions in phase II. In addition, more sessions were stopped in the C+SP group than in the SP group (11% vs 2% in phase I, and 20% vs 11% in phase II). However, no serious adverse events were recorded. The number of physiotherapy sessions not performed was similar in the two groups.

**Fig. 4:**
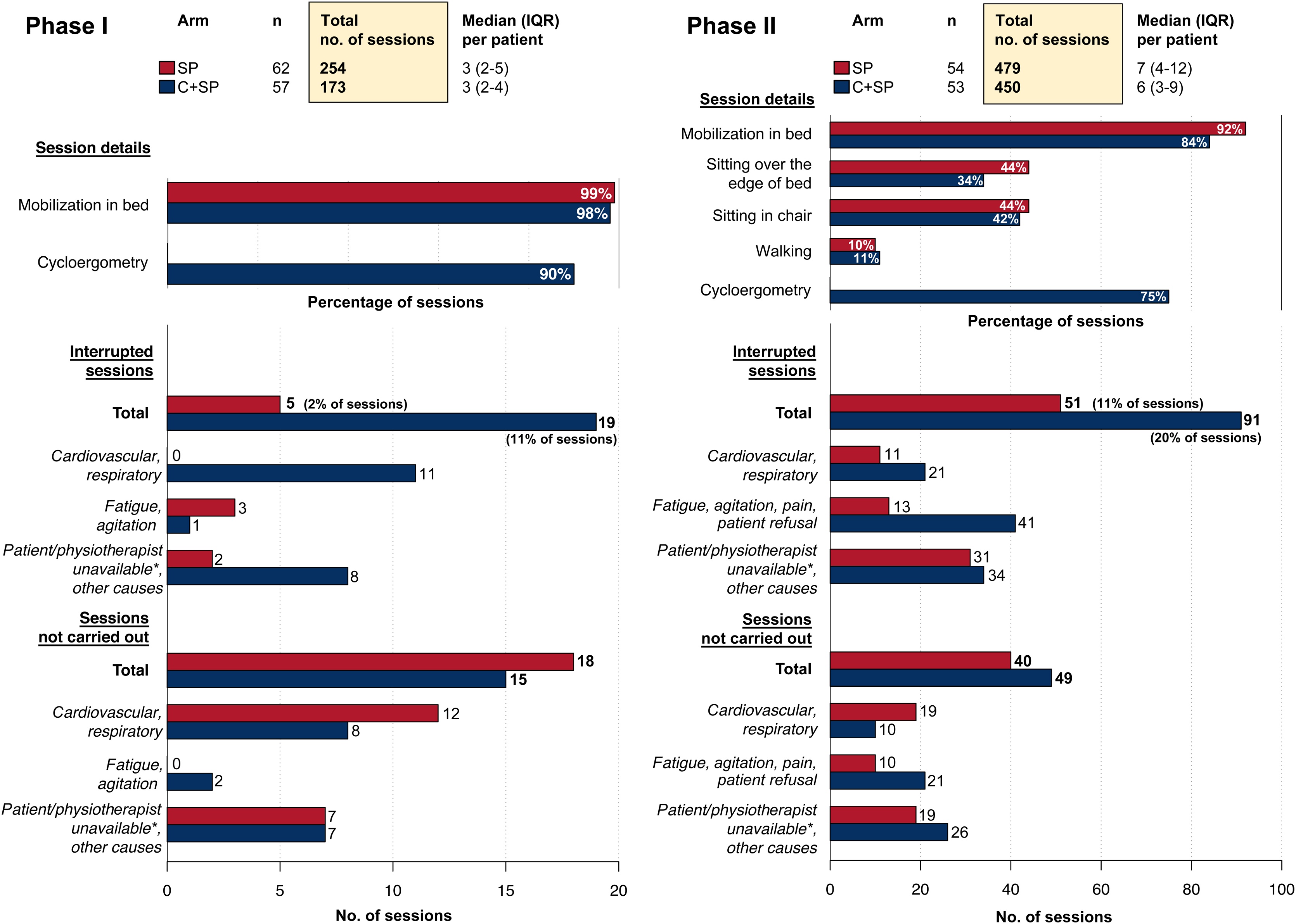
Physiotherapy sessions. Details of physiotherapy activities, reasons for stopping or not performing sessions in phase I and in phase II and by randomization arm. SP = standard physiotherapy. C+SP = cycloergometry + standard physiotherapy. m = median. IQR = interquartile range. * Patient/physiotherapist unavailable due to exams, nursing care, surgery, work overload, discharge or unknown reason.

The details of the sessions (mobilizations, sitting over the edge of the bed, sitting in a chair, walking, and cycling) and the specific reasons for stopping or not performing sessions are presented in Fig. 4 and Supplementary Table S5. It was not possible to distinguish the type of activity (standard physiotherapy or cycloergometry) when the sessions were stopped.

### ICU discharge conditions

Respiratory support, including non-invasive ventilation, tracheotomy, and high-flow or conventional oxygen, was recorded at ICU discharge. Respiratory support was received by 20% of patients in the SP group compared to 57% of patients in the C+SP group in phase I and/or in phase II. Moreover, a tracheotomy was performed in 28% of patients in the SP group compared to 6.2% of patients in the C+SP group in phase I and/or in phase II. Patients were discharged from ICU to an intermediate care unit (i.e. a Step Down Unit) or post-ICU Rehabilitation (44% of patients in the SP group compared to 27.5% of patients in the C+SP group, in phase I and/or in phase II). The other patients were discharged to a general ward (52% of patients in the SP group compared to 66% of patients in the C+SP group, in phase I and/or in phase II) (Supplementary Table S6).

### Mortality

We fitted a Cox proportional hazard regression with arms at first and second randomization as a time-dependent covariate. We found no evidence of an association between the use of cycloergometry and mortality (HR = 0.82; 95% CI 0.35–1.93, p = 0.6) (Fig. 2A, B, C).

## Discussion

The main result of this multicenter randomized controlled trial was that the addition of cycloergometry to early standard physiotherapy in sedated-ventilated patients admitted to ICU for septic shock was not significantly associated with a decrease in ICU LOS. However, in post-hoc analysis, the duration of mechanical ventilation was significantly shorter for patients who received cycloergometry (in phase I and/or in phase II) in addition to standard physiotherapy. To our knowledge, this is the first study to evaluate the effect of using early, intense and prolonged mobilization with cycloergometry in patients with septic shock ventilated in ICU.

These results are consistent with previous studies. A study published in 2018 by Fossat et al. [13] analyzed the effect of cycloergometry combined with electrostimulation in 314 patients in ICU. However, the characteristics of their patients were less severe (a SAPS II score of 47) and only 80% were ventilated, which was very different to the patients in our study. These authors did not find a significant difference in functional scores (MRC, ICU Mobility) at ICU discharge or at 6 months. Moreover, a recent international multicenter randomized controlled trial (RCT), evaluating early rehabilitation in 360 severe intubated-ventilated patients, found no evidence regarding their primary criteria (PFIT-s at 3 days after ICU discharge) or other outcomes (ICU LOS, MV duration), possibly related to the low number of cycling sessions in their study (a median of 3 [IQR: 2–5]).

These results are different to those of recent systematic reviews and meta-analyses that focused on global rehabilitation in intensive care. A systematic review of only RCTs with early inclusion (within 3 days after ICU admission or intubation) showed a significant difference in ICU LOS in favor of systematic early mobilization versus standard early mobilization [23]. Finally, in 2024, Schaller et al. in a systematic review with network meta-analysis [24] suggested that early mobilization alone could substantially shorten hospital LOS. In our study early physiotherapy was performed in the two groups, which could explain the absence of difference in ICU LOS. Anekwe et al. [25] analyzed nine RCTs focusing on early mobilization in adults admitted to ICU. These authors suggested that early rehabilitation compared to usual care reduced the risk of developing ICU-AW. A subgroup analysis confirmed that rehabilitation undertaken very early (<72 h from admission) protected against the development of ICU-AW, which is a major risk factor of prolonged ventilation. Early mobilization could be linked to a shorter duration of invasive MV in our study.

Conversely and more recently, an international multicenter RCT [26], compared early mobilization and usual care, in 750 patients. Authors reported similar data in the two groups regarding the number of ventilator-free days and ICU-free days at day 28.

Concerning studies evaluating specifically the addition of cycloergometry, a very recent meta-analysis [27] showed that cycling improved several criteria, including ICU LOS, physical function at ICU discharge and post-hospital discharge. This meta-analysis included 3274 patients with varied profiles: rehabilitation undertaken late in certain protocols (i.e. >7 days), respiratory status (inclusion of intubated or non-intubated patients), heterogeneous initial severity scores (SAPS or APACHE), etc. An older study [11] showed that in addition to standard physiotherapy, five weekly 20-minute cycloergometer sessions improved distance in the 6MWT at hospital discharge as well as quadriceps strength and self-reported functional status. Physiotherapy was started very late in this study (the mean ICU LOS before inclusion was 10 ± 8 or 14 ± 10 days depending on the group). In our study, physiotherapy was started very early and included cycloergometry in the interventional group. The fact that we were unable to demonstrate a difference in functional scores at ICU discharge could be linked to the initial severity of our patients and a lack of power.

Kagan et al. [28] studied the effect of cycloergometry and enriched nutrition in a small initially intubated-ventilated sample (n = 41). Authors did not show a significant difference in the duration of ventilation, mortality or LOS. Regarding SOFA and APACHE II scores, the population in this study was less severe than in our study. In terms of ICU LOS and ICU discharge conditions, our study has shown positive trends when comparing patients who received cycloergometry during at least one phase and patients who received no cycloergometry.

In 2018, Eggman et al. [29] evaluated 115 patients with an expected duration of mechanical ventilation of more than 72 h and assessed the effect of standardized early mobilization (including cycloergometry). These authors proposed very early physiotherapy (<48 h from admission). No difference was noted for functional scores (6MWT and Barthel) at ICU or hospital discharge, like in our study, or at 6 months. Overall, in their study, the median duration of mechanical ventilation was less than 5.4 days and the median ICU LOS was less than 6.6 days versus a median of 13–20 days in our study.

### Adverse events / not performed sessions

Contrary to several studies [[11], [12], [13],^29^,^30^], we observed a higher number of sessions that were not performed or stopped in the C+SP group. However, this observation is similar to that of a recent RCT comparing early mobilization and usual care, in which a high number of adverse events were reported in the early mobilization group [26]. We can consider that cycling is safe but patients require pain relief (37% of our patients were admitted for urgent surgery, particularly abdominal surgery) to reduce session anxiety and improve compliance.

### Limitations of the study

The main limitation of the study is that it failed to include the planned number of patients, resulting in lower precision in the outcome estimates (wider confidence intervals) and a reduced ability to detect a true association. Consequently, this study did not provide the expected level of evidence. Nevertheless, the two randomizations allowed a separate analysis of the two phases although the subgroups generated were small. Furthermore, reviewers were neither blinded nor external to the study, which may have influenced evaluation of muscle and functional scores. The absence of a strict protocol for extubation and discharge from the intensive care unit may have contributed to variations in the duration of mechanical ventilation and length of stay between centers, regardless of the specific effects of the intervention. In response to this absence of objective criteria, we strongly recommend following current guidelines.

### Strengths of the study

The major strength of the study is that it included patients from eight centers admitted to ICU with septic shock, requiring sedation and mechanical ventilation. This homogeneity in the severity of patients is rarely found in the literature [28,29]. This study constitutes the third largest cohort [13,14] on this subject of research (119 patients included) with a homogenous distribution of patients between urgent medical and surgical admission. Physiotherapy sessions with or without cycloergometry were started very early (a median of 2 days) in the two groups. Cycloergometry was proposed throughout ICU stay, including in phase I when patients were sedated. Finally, this study reports feasibility and safety data in a large cohort and we observed a high level of compliance and tolerance regarding the sessions (standard physiotherapy and cycloergometry).

## Conclusion

This multicenter randomized controlled trial failed to show any statistically significant evidence that cycloergometry and standard physiotherapy reduced ICU LOS, while confirming the safety of cycloergometry in patients with this level of therapeutic intensity. However, a trend was observed for a shorter median stay in ICU in patients who received cycloergometry and standard physiotherapy after awakening, supporting a need for further research on cycloergometry and early mobilization in patients with septic shock in intensive care unit.

## Muevelo study group

Bordeaux Pessac: Antoine Dewitte, MD, PhD; Orléans: Thierry Boulain, MD; Bordeaux: Olivier Guisset, MD, PhD; Suresnes: Matthieu Reffienna, DPT; Nantes: Marion Fresco, MD; Rouen: Nicolas Duquesne, DPT.

## CRediT authorship contribution statement

BM and GB contributed equally. Concept and design were performed by GB, BM, AA. Analysis and main drafting of the manuscript: BM, GB, VH. Drafting the manuscript for important intellectual content: all authors. Facilitation of inclusions was performed by the authors and their colleagues.

## Consent for publication

Not applicable.

## Ethical approval and consent to participate

The local Institutional Review Board (CPP Nord-Ouest 1; May 3, 2016) approved the study protocol. ClinicalTrials.gov (NCT02872792) and RCB number (2016-A00060-51).

All recruited patients or their legal representative provided written informed consent and all trial procedures conformed to local policies.

## Funding

The authors report grants from the French Ministry of Health (PHRIP 2014/211/HP). The funders of the study had no role in study design, data collection, data analysis, data interpretation, or writing of the report.

## Availability of supporting data

The datasets used and/or analysed during the current study are available from the corresponding author on reasonable request.

## Declaration of competing interest

The authors declare that they have no competing interests
