# Supplementary material for: Early mobilization with or without cycloergometry in patients with septic shock in Intensive Care Unit: a randomized controlled trial
Source: Ann Intensive Care. 2026 Feb 20;16:100034. doi: 10.1016/j.aicoj.2026.100034 (PMC13045550; doi:10.1016/j.aicoj.2026.100034)
Supplement: Supplementary file 1 [file mmc1.docx]

**Supplementary table 1**

|  |  | SP | C+SP |
| --- | --- | --- | --- |
|  |  | **n=62** | **n=57** |
| Demography |  |  |  |
| Age (yr) | *Mean (SD)* | 65.7 (15.3) | 64.7 (10.2) |
| Sex | *Male* | 40 (65%) | 36 (63%) |
|  | *Female* | 22 (35%) | 21 (37%) |
| Weight (kg) | *Mean (SD)* | 77.7 (20.8) | 77.1 (17.3) |
| BMI (kg/cm²) | *Mean (SD)* | 26.98 (6.12) | 27.06 (5.57) |
| Medical history |  |  |  |
| Cardiovascular disease |  | 37 (60%) | 33 (58%) |
| Cancer or autoimmune disease |  | 25 (40%) | 13 (23%) |
| Diabetes |  | 16 (26%) | 14 (25%) |
| Pulmonary disease |  | 11 (18%) | 6 (11%) |
| Chronic renal failure |  | 3 (4.8%) | 2 (3.5%) |
| Liver cirrhosis |  | 3 (4.8%) | 2 (3.5%) |
| Hemodynamics and biochemistry at inclusion | |  |  |
| SBP (mmHg) | *Median (IQR)* | 117 (107-129) | 112 (103-119) |
| DBP (mmHg) | *Median (IQR)* | 59 (53-69) | 59 (55-63) |
| MBP (mmHg) | *Median (IQR)* | 78 (72-86) | 75 (71-85) |
| SaO2 (%) | *Median (IQR)* | 98.0 (96.0-98.8) | 97.5 (96.3-98.7) |
| FIO2 (%) | *Median (IQR)* | 40 (35-50) | 40 (30-50) |
| PaO2 (kPa) | *Median (IQR)* | 11.13 (10.00-12.50) | 11.60 (10.10-14.10) |
| PaCO2 (kPa) | *Median (IQR)* | 5.0 (4.6-5.5) | 4.8 (4.1-5.5) |
| HCO3^-^ (mmol/L) | *Median (IQR)* | 24.2 (20.2-26.5) | 21.3 (18.5-23.6) |
| pH | *Median (IQR)* | 7.41 (7.36-7.44) | 7.37 (7.32-7.45) |
| Lactates (mmol/L) | *Median (IQR)* | 1.75 (1.58-2.90) | 2.00 (1.40-3.15) |
| Admission to hospital and ICU | |  |  |
| Type of admission | Scheduled surgery | 7 (11%) | 5 (8.8%) |
|  | Urgent surgery | 21 (34%) | 23 (40%) |
|  | Medical | 34 (55%) | 29 (51%) |
| Site of infection | Pulmonary | 31 (50%) | 22 (39%) |
|  | Abdominal | 25 (40%) | 29 (51%) |
|  | Urinary tract | 3 (4.8%) | 5 (8.8%) |
|  | Other | 3 (4.8%) | 1 (1.8%) |
| Time between admission to ICU and hemodynamic stability | Less than 12h | 3 (4.8%) | 6 (11%) |
|  | 12 to 24h | 20 (32%) | 12 (21%) |
|  | 24 to 48h | 22 (35%) | 28 (49%) |
|  | 48h to 72h | 17 (27%) | 11 (19%) |
| SAPS II score at admission in ICU | *mean (SD)* | 59.5 (19.7) | 59.0 (17.7) |
| SOFA score at admission to ICU | *median (IQR)* | 10 (7-13) | 9 (7-11) |
| RASS score before randomization | -2 | 3 (4.8%) | 1 (1.8%) |
|  | -3 | 16 (26%) | 7 (12%) |
|  | -4 | 19 (31%) | 17 (30%) |
|  | -5 | 23 (37%) | 31 (54%) |
|  | Unknown | 1 (1.6%) | 1 (1.8%) |
