# Supplementary material for: Early mobilization with or without cycloergometry in patients with septic shock in Intensive Care Unit: a randomized controlled trial
Source: Ann Intensive Care. 2026 Feb 20;16:100034. doi: 10.1016/j.aicoj.2026.100034 (PMC13045550; doi:10.1016/j.aicoj.2026.100034)
Supplement: Supplementary file 2 [file mmc2.docx]

**Supplementary table 2**

|  |  | SP | | C+SP | |
| --- | --- | --- | --- | --- | --- |
|  |  | **n=54** | | **n=53** | |
| Demography |  |  | |  | |
| Age (yr) | *Mean (SD)* | 65.1 (12.5) | | 65.1 (13.1) | |
| Sex | *Male* | 35 (65%) | | 36 (68%) | |
|  | *Female* | 19 (35%) | | 17 (32%) | |
| Weight (kg) | *Mean (SD)* | 78.9 (21.1) | | 75.9 (17.8) | |
| BMI (kg/cm²) | *Mean (SD)* | 27.34 (6.18) | | 26.54 (5.74) | |
| Medical history |  |  | |  | |
| Cardiovascular disease |  | 33 (61%) | | 31 (58%) | |
| Cancer or autoimmune disease |  | 18 (33%) | | 16 (30%) | |
| Diabetes |  | 16 (30%) | | 12 (23%) | |
| Pulmonary disease |  | 9 (17%) | | 7 (13%) | |
| Chronic renal failure |  | 3 (5.6%) | | 1 (1.9%) | |
| Liver cirrhosis |  | 2 (3.7%) | | 2 (3.8%) | |
| Hemodynamics and biochemistry at 2nd randomization | | | | |  |
| SaO2 (%) | *Median (IQR)* | 97.5 (96.3-98.4) | | 97.2 (96.5-98.2) | |
| FIO2 (%) | *Median (IQR)* | 35 (30-40) | | 30 (30-40) | |
| PaO2 (kPa) | *Median (IQR)* | 11.05 (9.72-12.77) | | 10.60 (9.40-12.10) | |
| PaCO2 (kPa) | *Median (IQR)* | 5.1 (4.6-5.7) | | 5.1 (4.7-5.6) | |
| HCO3^-^ (mmol/L) | *Median (IQR)* | 26.2 (23.5-29.9) | | 25.8 (23.4-28.8) | |
| pH | *Median (IQR)* | 7.44 (7.40-7.48) | | 7.44 (7.41-7.48) | |
| Lactates (mmol/L) | *Median (IQR)* | 1.40 (1.10-1.90) | | 1.50 (1.19-2.03) | |
| Scores at 2^nd^ randomization | | |  | |  |
| SOFA score | *Median (IQR)* | 6.5 (4.0-9.8) | | 7.0 (5.0-10.0) | |
| RASS score | 1 | 0 | | 4 (7.5%) | |
|  | -0 | 37 (69%) | | 23 (43%) | |
|  | -1 | 13 (24%) | | 24 (45%) | |
|  | -3 | 3 (5.6%) | | 1 (1.9%) | |
|  | Unknown | 1 (1.9%) | | 1 (1.9%) | |
