# Supplementary material for: Early mobilization with or without cycloergometry in patients with septic shock in Intensive Care Unit: a randomized controlled trial
Source: Ann Intensive Care. 2026 Feb 20;16:100034. doi: 10.1016/j.aicoj.2026.100034 (PMC13045550; doi:10.1016/j.aicoj.2026.100034)
Supplement: Supplementary file 3 [file mmc3.docx]

**Supplementary table 3**

|  |  | **n** | **Subdistribution hazard ratio (95% CI)** | **p-value** |
| --- | --- | --- | --- | --- |
| **Phase I: probability of first awakening from hemodynamic stability** | | | | |
| **SOFA score at inclusion** | | | |  |
|  | < 10 | 49 | --- |  |
|  | ≥ 10 | 70 | 0.67 (0.48-0.94) | 0.021 |
| **Type of mobilization in phase I** | |  | |  |
|  | SP | 62 | --- |  |
|  | C+SP | 57 | 1.10 (0.79-1.54) | 0.6 |
|  |  |  |  |  |
| **Phase II: probability of ICU discharge from first awakening** | | | | |
| **SOFA score at first awakening** | | | |  |
|  | < 10 | 64 | --- |  |
|  | ≥ 10 | 25 | 0.50 (0.28-0.91) | 0.022 |
| **MRC score at first awakening** | | | |  |
|  | Less than 24 | 25 | --- |  |
|  | 24 to 47 | 42 | 1.77 (1.00-3.13) | 0.49 |
|  | 48 or more | 22 | 3.55 (1.94-6.48) | <0.0001 |
| **Type of mobilization in phase I** | | | | |
|  | SP | 47 | --- |  |
|  | C+SP | 42 | 1.24 (0.74-2.08) | 0.4 |
| **Type of mobilization in phase II** | |  |  |  |
|  | SP | 43 | --- |  |
|  | C+SP | 46 | 1.50 (0.83-2.72) | 0.18 |
