# Supplementary material for: Early mobilization with or without cycloergometry in patients with septic shock in Intensive Care Unit: a randomized controlled trial
Source: Ann Intensive Care. 2026 Feb 20;16:100034. doi: 10.1016/j.aicoj.2026.100034 (PMC13045550; doi:10.1016/j.aicoj.2026.100034)
Supplement: Supplementary file 4 [file mmc4.docx]

**Supplementary table 4**

|  |  | | | Total | | SP | | C+SP | | p-value | |
| --- | --- | --- | --- | --- | --- | --- | --- | --- | --- | --- | --- |
|  |  | | | **n=91** | | **n=45** | | **n=46** | |  | |
| Muscle strength (MRC score) | | | |  | |  | |  | |  | |
| At first awakening | | | |  | |  | |  | |  | |
|  | *No. reported scores* | | | 79 | | 36 | | 43 | |  | |
|  | *Median (IQR)* | | | 35 (23-48) | | 31 (20-46) | | 36 (28-48) | | 0.4 | |
| Patients with SP in phase I | | | | 36 (26-46) | | 36 (26-46) | | 34 (24-45) | |  | |
| Patients with C+SP in phase I | | | | 32 (20-48) | | 32 (20-48) | | 24 (19-46) | |  | |
|  | < 24 | | | 20 (25%) | | 12 (33%) | | 8 (19%) | |  | |
|  | 24 to 47 | | | 38 (48%) | | 15 (42%) | | 23 (53%) | |  | |
|  | ≥ 48 | | | 21 (27%) | | 9 (25%) | | 12 (28%) | |  | |
| At ICU discharge | | | |  | |  | |  | |  | |
|  | *No. reported scores* | | | 79 | | 40 | | 39 | |  | |
|  | *Median (IQR)* | | | 48 (40-55) | | 48 (38-54) | | 48 (42-56) | | 0.2 | |
|  | < 24 | | | 4 (5.1%) | | 3 (7.5%) | | 1 (2.6%) | |  | |
|  | 24 to 47 | | | 30 (38%) | | 16 (40%) | | 14 (36%) | |  | |
|  | ≥ 48 | | | 45 (57%) | | 21 (52%) | | 24 (62%) | |  | |
| Score gain per patient | |  |  | |  | |  | |  | |  |
| *No. measurable gains* | | | | 72 | | 33 | | 39 | |  | |
|  | *Median (IQR)* | | | 12 (2-20) | | 13 (2-20) | | 9 (3-19) | | 0.9 | |
|  | < 0 | | | 6 (8.3%) | | 3 (9.1%) | | 3 (7.7%) | |  | |
|  | 0 to 9 | | | 28 (39%) | | 11 (33%) | | 17 (44%) | |  | |
|  | 10 to 19 | | | 17 (24%) | | 7 (21%) | | 10 (26%) | |  | |
|  | ≥ 20 | | | 21 (29%) | | 12 (36%) | | 9 (23%) | |  | |
| Physical function (PFIT score) | | | |  | |  | |  | |  | |
| At first awakening | | | |  | |  | |  | |  | |
|  | *No. reported scores* | | | 69 | | 31 | | 38 | |  | |
|  | *Median (IQR)* | | | 2 (0-4) | | 2 (0-4) | | 2 (1-5) | | 0.17 | |
| Patients with SP in phase I | | | | 2 (1-4) | | 2 (1-4) | | 2 (0-4) | |  | |
| Patients with C+SP in phase I | | | | 2 (0-4) | | 2 (0-4) | | 1 (0-2) | |  | |
|  | 0 | | | 20 (29%) | | 12 (39%) | | 8 (21%) | |  | |
|  | 1 to 4 | | | 33 (48%) | | 14 (45%) | | 19 (50%) | |  | |
|  | 5 or more | | | 16 (23%) | | 5 (16%) | | 11 (29%) | |  | |
| At ICU discharge | | | |  | |  | |  | |  | |
|  | *No. reported scores* | | | 70 | | 35 | | 35 | |  | |
|  | *Median (IQR)* | | | 7 (4-8) | | 6 (4-8) | | 7 (4-9) | | 0.5 | |
|  | 0 | | | 4 (5.7%) | | 3 (8.6%) | | 1 (2.9%) | |  | |
|  | 1 to 4 | | | 17 (24%) | | 7 (20%) | | 10 (29%) | |  | |
|  | 5 or more | | | 49 (70%) | | 25 (71%) | | 24 (69%) | |  | |
| Score gain per patient | | | |  | |  | |  | |  | |
| *No. measurable gains* | | | | 61 | | 28 | | 33 | |  | |
|  | *Median (IQR)* | | | 4 (2-6) | | 5 (2-6) | | 4 (2-5) | | > 0.9 | |
|  | < 0 | | | 12 (20%) | | 6 (21%) | | 6 (18%) | |  | |
|  | 1 to 2 | | | 8 (13%) | | 3 (11%) | | 5 (15%) | |  | |
|  | 3 to 5 | | | 24 (39%) | | 10 (36%) | | 14 (42%) | |  | |
|  | 6 or more | | | 17 (28%) | | 9 (32%) | | 8 (24%) | |  | |
