# Supplementary material for: Early mobilization with or without cycloergometry in patients with septic shock in Intensive Care Unit: a randomized controlled trial
Source: Ann Intensive Care. 2026 Feb 20;16:100034. doi: 10.1016/j.aicoj.2026.100034 (PMC13045550; doi:10.1016/j.aicoj.2026.100034)
Supplement: Supplementary file 5 [file mmc5.docx]

**Supplementary table 5**

| **Phase I (from hemodynamic stability to first awakening) – n=119** | | **SP** | **C+SP** |
| --- | --- | --- | --- |
|  |  | **n=62** | **n=57** |
| **Time from ICU admission to the first physiotherapy session**  **Sessions performed** | Median (IQR) per patient  Median (IQR) per patient | **2 (2-3)**  **3 (2-5)** | **2 (2-3)**  **3 (2-4)** |
|  | *Total no. of sessions* | *254* | *173* |
| **Session details** | *% of patients who performed the activity at least once* | | |
|  | Mobilization in bed | 97% | 95% |
|  | Cycloergometry | - | 91% |
| **Interrupted sessions** | ***Total no. of patients*** | **3** | **13** |
| Reasons for interruption | Cardiovascular, respiratory | 0 | 7 |
|  | Fatigue, agitation | 2 | 1 |
|  | Patient or PT unavailable*,  Other causes | 1 | 6 |
| **Sessions not carried out** | ***Total no. of distinct patients*** | **10** | **9** |
| Reason for session not carried out | Cardiovascular, respiratory | 7 | 4 |
|  | Fatigue, agitation | 0 | 2 |
|  | Patient or PT unavailable*,  Other causes | 5 | 5 |
| **Phase II (from first awakening to ICU discharge) – n=107** | | **SP** | **C+SP** |
|  |  | **n=54** | **n=53** |
| **Sessions performed** | Median (IQR) per patient | **7 (4-12)** | **6 (3-9)** |
|  | *Total no. of sessions* | *479* | *450* |
| **Session details** | *% of patients who performed the activity at least once* | | |
|  | Mobilization in bed | 98% | 96% |
|  | Sitting over the edge of the bed | 76% | 78% |
|  | Sitting in a chair | 78% | 81% |
|  | Walking | 33% | 37% |
|  | Cycloergometry | - | 98% |
| **Interrupted sessions** | ***Total no. of patients*** | **18** | **31** |
| Reasons for interruption | Cardiovascular, respiratory | 7 | 11 |
|  | Fatigue, agitation, pain, patient refusal | 6 | 18 |
|  | Patient or PT unavailable*,  Other causes | 14 | 21 |
| **Sessions not carried out** | ***Total no. of patients*** | **22** | **19** |
| Reason for session not carried out | Cardiovascular, respiratory | 13 | 9 |
|  | Fatigue, agitation, pain, patient refusal | 6 | 9 |
|  | Patient or PT unavailable*,  Other causes | 15 | 10 |
