# Supplementary material for: Early mobilization with or without cycloergometry in patients with septic shock in Intensive Care Unit: a randomized controlled trial
Source: Ann Intensive Care. 2026 Feb 20;16:100034. doi: 10.1016/j.aicoj.2026.100034 (PMC13045550; doi:10.1016/j.aicoj.2026.100034)
Supplement: Supplementary file 6 [file mmc6.docx]

**Supplementary table 6**

|  | Total | SP only | C+SP in phase I and/or in phase II |  |
| --- | --- | --- | --- | --- |
|  | **n=90** | **n=25** | **n=65** | **p-value** |
| ICU discharge destination |  |  |  | **0.5** |
| General ward | 56 (62%) | 13 (52%) | 43 (66%) |  |
| Intermediate care unit | 27 (30%) | 10 (40%) | 17 (26%) |  |
| Post-ICU Rehabilitation | 2 (2.2%) | 1 (4.0%) | 1 (1.5%) |  |
| Home | 1 (1.1%) | 0 | 1 (1.5%) |  |
| Other | 4 (4.4%) | 1 (4.0%) | 3 (4.6%) |  |
| ICU discharge conditions |  |  |  | **0.0006** |
| Without respiratory support | 42 (47%) | 5 (20%) | 37 (57%) |  |
| Conventional oxygen | 30 (33%) | 13 (52%) | 17 (26%) |  |
| High-flow oxygen | 6 (6.7%) | 0 | 6 (9.2%) |  |
| NIV | 1 (1.1%) | 0 | 1 (1.5%) |  |
| Ventilated tracheotomy | 10 (11%) | 6 (24%) | 4 (6.2%) |  |
| Non-ventilated tracheotomy | 1 (1.1%) | 1 (4.0%) | 0 |  |
