# Supplementary material for: Early mobilization with or without cycloergometry in patients with septic shock in Intensive Care Unit: a randomized controlled trial
Source: Ann Intensive Care. 2026 Feb 20;16:100034. doi: 10.1016/j.aicoj.2026.100034 (PMC13045550; doi:10.1016/j.aicoj.2026.100034)
Supplement: Supplementary file 7 [file mmc7.pdf]

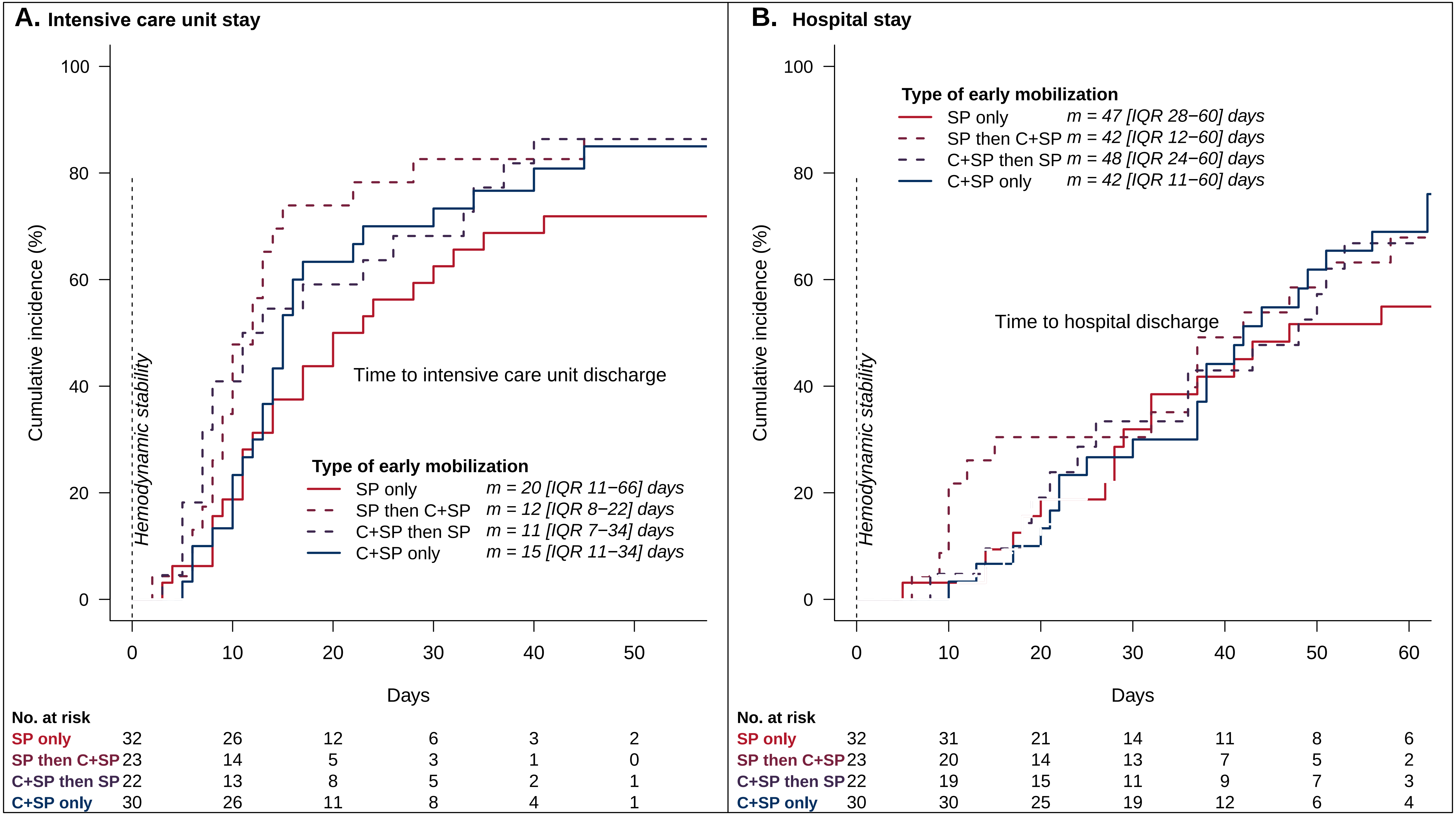
A. Intensive care unit stay
Cumulative incidence (%)
100
80
60
40
20
0
Hemodynamic stability
Time to intensive care unit discharge
Type of early mobilization
SP only m = 20 [IQR 11−66] days
SP then C+SP m = 12 [IQR 8−22] days
C+SP then SP m = 11 [IQR 7−34] days
C+SP only m = 15 [IQR 11−34] days
0 10 20 30 40 50
Days
No. at risk
SP only 32 26 12 6 3 2
SP then C+SP 23 14 5 3 1 0
C+SP then SP 22 13 8 5 2 1
C+SP only 30 26 11 8 4 1
B. Hospital stay
Cumulative incidence (%)
100
80
60
40
20
0
Type of early mobilization
SP only m = 47 [IQR 28−60] days
SP then C+SP m = 42 [IQR 12−60] days
C+SP then SP m = 48 [IQR 24−60] days
C+SP only m = 42 [IQR 11−60] days
Hemodynamic stability
Time to hospital discharge
0 10 20 30 40 50 60
Days
No. at risk
SP only 32 31 21 14 11 8 6
SP then C+SP 23 20 14 13 7 5 2
C+SP then SP 22 19 15 11 9 7 3
C+SP only 30 30 25 19 12 6 4
