# Supplementary figures and images for: Early mobilization with or without cycloergometry in patients with septic shock in Intensive Care Unit: a randomized controlled trial
Source: Ann Intensive Care. 2026 Feb 20;16:100034. doi: 10.1016/j.aicoj.2026.100034 (PMC13045550; doi:10.1016/j.aicoj.2026.100034)

Time to end of invasive mechanical ventilation

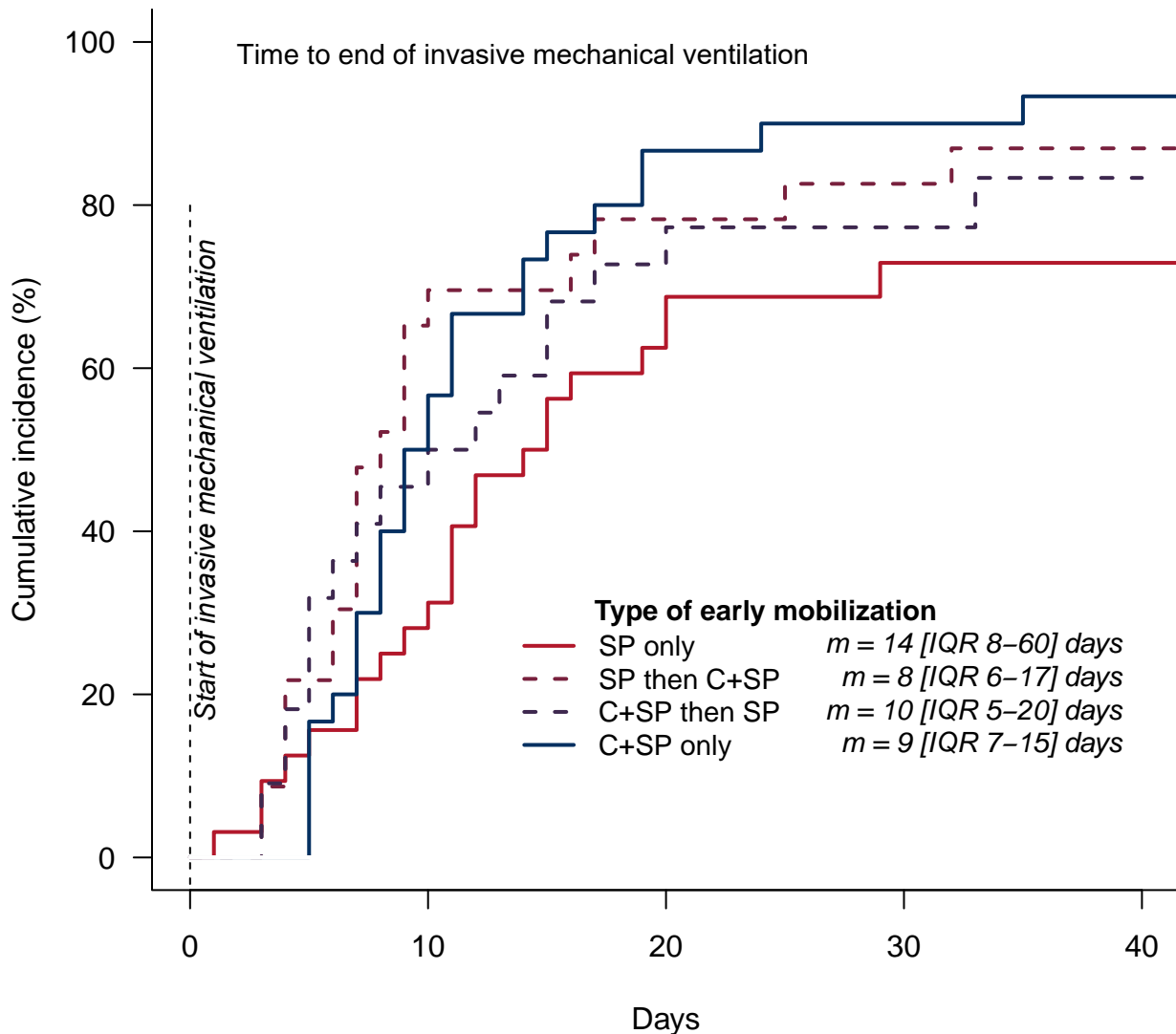

No. at risk

|              |    |    |    |   |   |
|--------------|----|----|----|---|---|
| SP only      | 32 | 23 | 11 | 5 | 3 |
| SP then C+SP | 23 | 8  | 4  | 3 | 1 |
| C+SP then SP | 22 | 12 | 5  | 3 | 1 |
| C+SP only    | 30 | 15 | 4  | 2 | 1 |

Supplement: Supplementary file 8 [file mmc8.pdf]

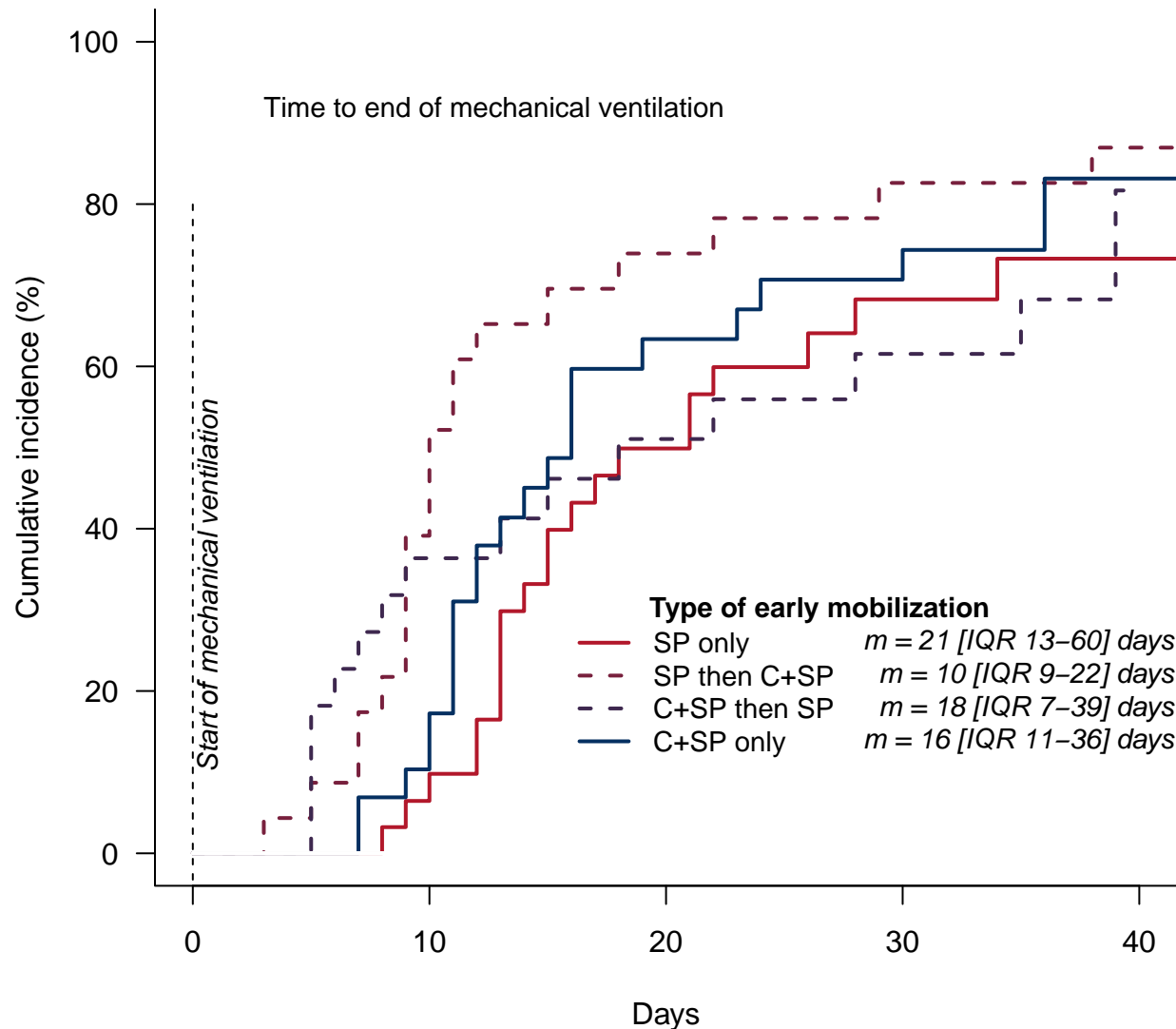

**No. at risk**

|                     |    |    |    |   |   |
|---------------------|----|----|----|---|---|
| <b>SP only</b>      | 32 | 28 | 14 | 6 | 3 |
| <b>SP then C+SP</b> | 23 | 14 | 5  | 3 | 1 |
| <b>C+SP then SP</b> | 22 | 14 | 9  | 6 | 1 |
| <b>C+SP only</b>    | 30 | 26 | 10 | 7 | 3 |

Supplement: Supplementary file 9 [file mmc9.pdf]
