# Supplementary material for: Early mobilization with or without cycloergometry in patients with septic shock in Intensive Care Unit: a randomized controlled trial
Source: Ann Intensive Care. 2026 Feb 20;16:100034. doi: 10.1016/j.aicoj.2026.100034 (PMC13045550; doi:10.1016/j.aicoj.2026.100034)
Supplement: Supplementary file 10 [file mmc10.pdf]

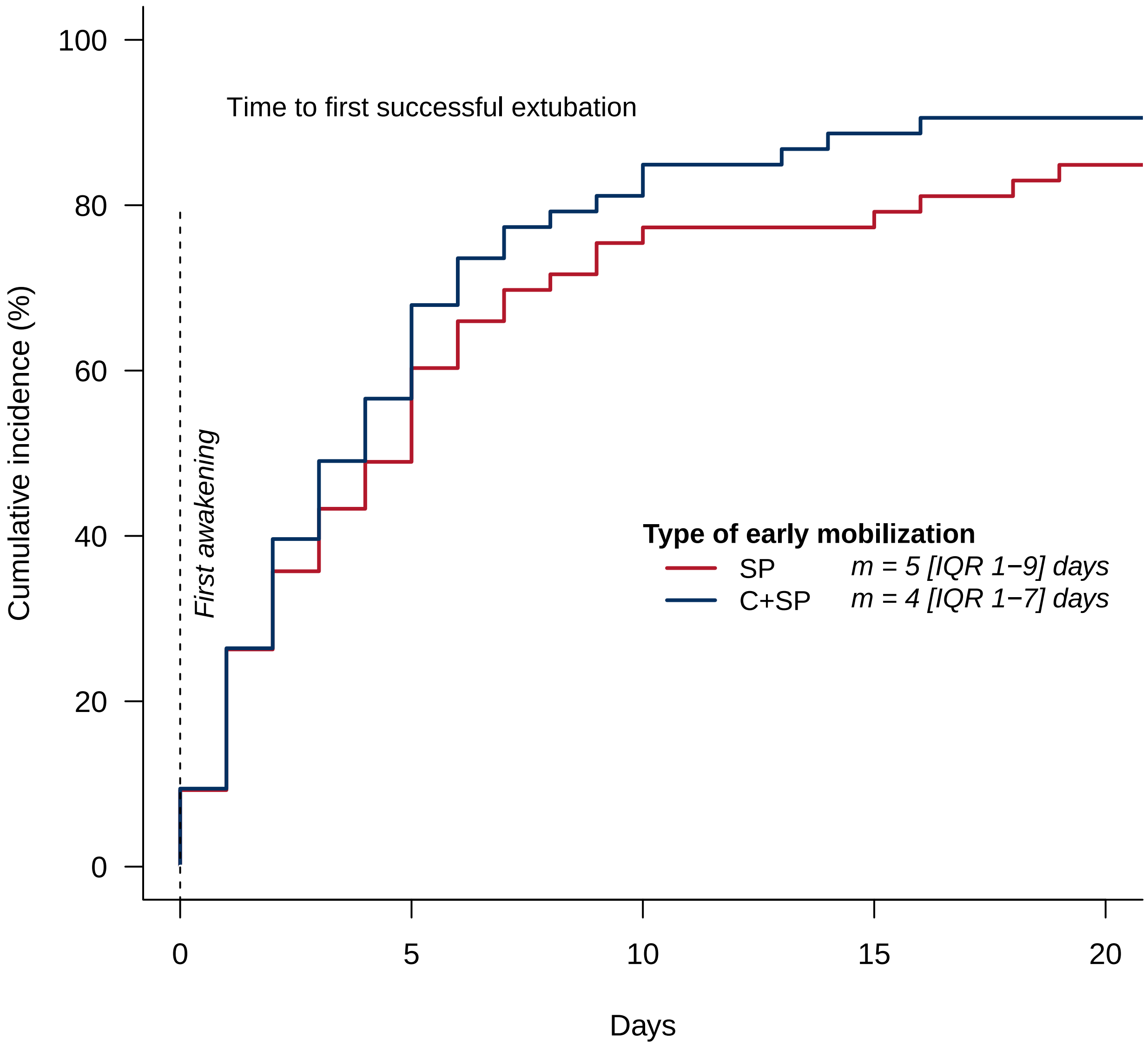

No. at risk

	0	5	10	15	20
**SP**	54	27	13	10	5
**C+SP**	53	23	9	4	3
